# Comparative Effects of the Single and Binary Fermentations of *Latilactobacillus sakei* and *Staphylococcus carnosus* on the Growth and Metabolomic Profiles of Fermented Beef Sausages

**DOI:** 10.3390/microorganisms13071523

**Published:** 2025-06-29

**Authors:** Xuan Li, Yangyi Zheng, Wenming Cui, Xueyuan Bai, Chaozhi Zhu, Gaiming Zhao

**Affiliations:** 1 Henan Key Lab of Meat Processing and Quality Safety Control, Henan Agricultural University, Zhengzhou 450002, China; lx04080503@163.com (X.L.);; 2 College of Food Science and Technology, Henan Agricultural University, Zhengzhou 450002, China

**Keywords:** lactic acid bacteria, fermented meat, non-volatile metabolic profiles, metabolomic pathways

## Abstract

*Latilactobacillus sakei* (*L. sakei*) and *Staphylococcus carnosus* (*S. carnosus*) are common starters for fermented sausages. However, the mechanism underlying the effects of these two microorganisms on co-cultivation in sausages remains unclear. This study compared the changes in metabolomics following fermentation by *L. sakei* and *S. carnosus* individually and in combination. After two days of fermentation, the pH values of the LS (*Latilactobacillus* Single), SC (*Staphylococcus* Single), and LSSC (*Latilactobacillus*-*Staphylococcus* Combined) groups were 4.59, 5.19, and 4.86. By comparing the common differential metabolites among the three groups, it was found that the content of N2-acetyl-L-ornithine decreased after single fermentation with *L. sakei*, while the content of N2-acetyl-L-ornithine increased after single fermentation with *S. carnosus* and combined fermentation with *L. sakei*. Additionally, KEGG pathway analysis identified eight key metabolic pathways, including purine metabolism, starch and sucrose metabolism. In addition, it was found that *L. sakei* produced D-Galactose during fermentation, which could be utilized by *S. carnosus*. The co-fermentation of *L. sakei* and *S. carnosus* promoted the production of D-sorbitol. Our results suggest that the metabolic interactions between *L. sakei* and *S. carnosus* increase the number of functional metabolites in co-fermented sausages. These findings provide valuable insights and new research directions for the study of LAB and CNS interactions, as well as for the development of fermentation agents.

## 1. Introduction

Fermented sausage is a meat product that undergoes microbial fermentation to develop its unique flavor. It is characterized by high nutritional value, distinct taste, and long shelf life. However, traditional natural fermentation has a long production cycle, unstable product quality, and potential safety risks. The use of artificially inoculated microbial starters can guide the fermentation process, inhibit the growth of pathogenic microorganisms, and prevent the synthesis of potentially harmful compounds such as biogenic amines, thereby ensuring the safety of the final product. Meanwhile, microbial starter cultures can also standardize product characteristics (including flavor and color), shorten the fermentation time, and produce products of the same quality throughout the year in any climate zone [1,2]. Nowadays, the meat industry has adopted starter culture groups that combine lactic acid bacteria (LAB), non-pathogenic coagulase-negative staphylococci (CNS), yeast, and mold cultures. The most commonly used bacterial starter cultures for developing fermented sausages are *Lactiplantibacillus plantarum* (*L. plantarum*), *Lacticaseibacillus casei* (*L. casei*), *Latilactobacillus sakei* (*L. sakei*), *Lactiplantibacillus pentosus* (*L. pentosus*), and *Pediococcus pentosaceus* (*P. pentosaceus*), as well as some species of *Staphylococcus*, such as *Staphylococcus xylosus* (*S. xylosus*) and *Staphylococcus carnosus* (*S. carnosus*) [3]. The lactic acid produced during the fermentation process of LAB acts on meat proteins, changing their water-holding capacity, thereby contributing to the texture, moisture, flavor, and aroma of the product, and simultaneously affecting its microbial safety. On the other hand, CNS plays an important role in the development of sensory characteristics of sausages by reducing nitrate, conferring them with a distinctive red color, and lipolytic and proteolytic activities [4]. Although LAB and CNS are usually involved in the fermentation process, it is difficult to guarantee the final quality of sausage products through independent fermentation. Some studies have reported that multi-strain probiotics make fermented sausages have more desirable flavor and color than single strains [5,6]. Therefore, there is growing interest in the co-fermentation of starter cultures and multi-strain probiotics in fermented sausages.

Metabolites can be used to evaluate the nutritional value, traceability, and authenticity, as well as the physiological functions of fermented meat products. The application of untargeted metabolomics plays a crucial role in clarifying the qualitative and quantitative changes of small-molecule metabolites in the metabolic process, providing a comprehensive understanding of the microbial metabolic process. Its uses extend to various fields, including the food industry [7]. The selection of starter cultures has a significant impact on the development of functional metabolites. Zhao et al. [8] found that mixed fermentation of *P. pentosaceus* and *S. carnosus* is more conducive to the accumulation of amino acids and derivatives that exhibit taste, particularly the aromatic amino acids l-glutamate and l-arginine. Wu et al. [9] found that the mixed fermentation of *Staphylococcus vitulinus* and *S. xylosus* improves the contents of peptides, organic acids, and amino acid derivatives in Chinese cured meat through purine metabolism, glutathione metabolism, and glutamate metabolism. At present, most studies focus on exploring the impact of compound bacteria on fermented meat products through metabolomic technology [10,11]. However, there is still a lack of studies using metabolomics to investigate the impact of microbial interactions on fermented sausages, especially at the fermentation stage.

*L. sakei* is primarily found in meat and can withstand challenging conditions such as high salt concentrations, low water activity, low temperatures, and low pH, making it widely used in fermented meat products [12]. In our previous study, *L. sakei* was isolated from Yunnan cured meat. The growth environment of *L. sakei* and *S. carnosus* (both individually and in combination) in fermented sausages was optimized based on inoculum size, fermentation temperature, and salt addition [13]. The primary aim of this research was to compare the growth and metabolomic characteristics of *L. sakei* and *S. carnosus* as single and binary probiotics, with the goal of identifying their interactions and patterns during fermentation. This study provides valuable and innovative insights into the microbial interactions occurring during fermentation. These findings not only enhance our fundamental understanding but also have significant potential to guide future strain selection and product improvements in the fermented meat industry.

## 2. Materials and Methods

### 2.1. Materials and Chemicals

*L. sakei* was isolated and screened from Yunnan cured meat; GIM 1.955 *S. carnosus* (Guangdong Microbial Culture Collection Center, Guangzhou, China); beef (knuckle) (Henan Yisai Beef Co., Ltd., Jiaozuo, China); pork fat (Zhutun farmers Market, Zhengzhou, China); the ingredients, including salt, sucrose, and 48–50 mm sausage casings (Changshen supermarket, Zhengzhou, China); sodium nitrite (Weifang Jada Chemical Technology Co., Ltd., Weifang, China); edible glucose (Weifang Shengtai Pharmaceutical Co., Ltd., Weifang, China); sodium erythorbate (Zhucheng Huayuan Biological Engineering Co., Ltd., Weifang, China).

### 2.2. Preparation and Sampling of Fermented Beef Sausages

Microbial preparation process: *L. sakei* was isolated from Yunnan cured meat. *L. sakei* and *S. carnosus* were individually grown in MRS broth (Qingdao Hi—Tech Industrial Park Hope Bio—Technology Co., Ltd. Qingdao, Shandong, China) and LB liquid medium (Qingdao Hi—Tech Industrial Park Hope Bio—Technology Co., Ltd. Qingdao, Shandong, China) at 37 °C for 12 and 14 h respectively, activated for 3 generations, inoculated into the medium, and cultivated at 37 °C for 12 h. Subsequently, 1% (v:v) of the bacterial suspension from the medium was introduced to the enrichment medium (MRS broth and LB liquid medium), the viable cell count was standardized to the required multiplicity and cultivated at 37 °C for 12 h. After centrifugation, the supernatant was discarded and rinsed 3 times with physiological saline, followed by the addition of sterile water for subsequent usage.

Sample Preparation: The groups were set up as follows: LS (*Latilactobacillus* Single) was prepared with beef (fat content 2%): pork fat (the ratio is 7:3, and the minced meat has a caliber of 5 mm), and then 0.5% glucose, 4% carbohydrates, 1.7% salt, 0.015% sodium nitrite, and 0.05% sodium erythorbate were added. After thorough stirring and mixing, the mixture was placed in a 4 °C cold storage environment and left to stand for 12 h. The mixed material was then removed and inoculated with *L. sakei* at an addition amount of 1.17 × 10^7^ CFU/g (7.1 lg CFU/g). The inoculated mixture was first fermented at 24 °C for 48 h.

SC (*Staphylococcus* Single) was made using beef: pork fat (the ratio is 7:3, and the minced meat has a caliber of 5 mm), and then 0.5% glucose, 4% carbohydrates, 2.05% salt, 0.015% sodium nitrite, and 0.05% sodium erythorbate were added. After thorough stirring and mixing, the mixture was placed in a 4 °C cold storage environment and left to stand for 12 h. The mixed material was then taken out and inoculated with *S. carnosus* at an additional amount of 1.58 × 10^7^ CFU/g (7.2 lg CFU/g). The inoculated mixture was first fermented at 29 °C for 48 h.

LSSC (*Latilactobacillus* -*Staphylococcus* Combined) was prepared by using beef: pork fat (the ratio is 7:3, and the minced meat has a caliber of 5 mm), and then 0.5% glucose, 4% carbohydrates, 2% salt, 0.015% sodium nitrite, and 0.05% sodium erythorbate were added. After thorough stirring and mixing, the mixture was placed in a 4 °C cold storage environment and left to stand for 12 h. The mixed material was then taken out and inoculated with *L. sakei* at an addition amount of 3.98 × 10^6^ CFU/g (6.6 lg CFU/g) and *S. carnosus* at an addition amount of 1.58 × 10^7^ CFU/g (7.2 lg CFU/g). The inoculated mixture was first fermented at 30 °C for 48 h.

As the strains were added after the curing process, samples on day 0 were collected during the curing period (after evenly blending the beef and the ingredients). Samples of the three groups were acquired at two distinct time points (0 and 48 h). All the samples were promptly put into sterile tubes and stored at −80 °C.

### 2.3. Enumeration of LAB and Staphylococci

A total of 10.0 g of the sample was accurately measured and added to a container containing 90.0 mL of sterile 0.9% physiological saline. The mixture was thoroughly homogenized by magnetic stirring to complete the initial dilution. According to the estimated microbial concentration of the sample, a 10-fold serial dilution method was employed to perform gradient dilution, yielding dilution solutions at optimal concentrations for detection. Then, 100 μL of the bacterial suspension at each dilution was evenly spread on the surface of MRS agar medium (Qingdao Hi-Tech Industrial Park Hope Bio-Technology Co., Ltd., Qingdao, China) for the selective cultivation of LAB, while an appropriate amount of the diluted suspension was inoculated on mannitol sodium chloride agar medium (Qingdao Hi-Tech Industrial Park Hope Bio-Technology Co., Ltd., Qingdao, China) for the selective isolation of staphylococci. The inoculated media were incubated at 37 °C for 48 h. After cultivation, plates with colony counts between 30 and 300 were selected, and the number of colony-forming units (CFU) was counted using a colony counter or manual counting. Finally, the cell concentration was expressed as colony-forming units per gram of fermented sausage meat (CFU/g) [14].

### 2.4. pH Analysis

A total of 5.0 g of the sample was weighed and placed in a beaker containing 50 mL of 0.1 mol/L potassium chloride solution. The sample was homogenized using a tissue homogenizer for 2 min to ensure thorough mixing between the sample and the solution. Subsequently, the calibrated electrodes of the pH meter (BPP-7800, BELL Analytical Instruments (Dalian) Co., Ltd., Dalian, China, 25 °C) were immersed in the sample solution, and the pH value was recorded. Each sample was measured in triplicate, and the average value was reported as the final test result [15].

### 2.5. Sample Preparation

A sample weighing 50 ± 5 mg was measured using an analytical balance and transferred into a 2 mL centrifuge tube. A 6 mm diameter grinding bead was added to the tube, followed by 400 μL of an extraction solution prepared by mixing methanol and water at a volume ratio of 4:1. This extraction solution was uniformly spiked with four internal standard substances, including L-2-chlorophenylalanine at a concentration of 0.02 mg/mL. The sample-loaded centrifuge tube was then placed in a cryogenic tissue grinder and subjected to grinding at an oscillation frequency of 50 Hz for 6 min under a low-temperature environment of −10 °C. After grinding, cryogenic ultrasonic extraction was performed at a temperature of 5 °C, a frequency of 40 KHz, and a duration of 30 min. Post-ultrasonication, the sample was allowed to stand at −20 °C for 30 min, followed by centrifugation at 13,000× *g* for 15 min at 4 °C. Once centrifugation was complete, the supernatant was transferred into a vial equipped with an inner insert, ready for instrumental analysis. Additionally, 20 μL of the supernatant from each sample was pooled to prepare a quality control (QC) sample, which was used for monitoring the quality of the experimental process.

### 2.6. LC-MS Detection

Chromatographic conditions: Separation and detection were carried out using an ACQUITY UPLC HSS T3 column (100 mm × 2.1 mm i.d., 1.8 μm; Waters, Milford, MA, USA). The mobile phase consisted of two components: mobile phase A was a mixture of 95% water and 5% acetonitrile containing 0.1% formic acid, while mobile phase B was a mixture of 47.5% acetonitrile, 47.5% isopropanol, and 5% water with 0.1% formic acid. The injection volume was set at 3 μL, and the column temperature was maintained at a constant 40 °C. The components of the samples were effectively separated through an optimized gradient elution program of the mobile phase.

Mass spectrometric conditions: The samples were ionized by an electrospray ionization (ESI) source, and mass spectrometry signals were acquired using positive and negative ion scanning modes, respectively. The specific parameters were set as follows: the scanning range was 70–1050 *m*/*z*; the sheath gas flow rate was 50 arb; the auxiliary gas flow rate was 13 arb; the heating temperature was controlled at 425 °C; the capillary temperature was maintained at 325 °C; the spray voltage was 3500 V in positive mode and −3500 V in negative mode; the S-Lens voltage was set to 50; the normalized collision energies were 20, 40, and 60 eV; the full MS resolution was 60,000, the MS2 resolution was 7500. These parameter settings ensured efficient detection and accurate analysis of sample ions [16].

### 2.7. Metabolite Identification

Raw data were imported into the metabolomics processing software Progenesis QI 2.0 (Waters Corporation, Milford, MA, USA) for operations including baseline filtering, peak identification, integration, retention time correction, and peak alignment, ultimately yielding a data matrix containing information such as retention time, mass-to-charge ratio, and peak intensity. Subsequently, the software was employed to perform database retrieval and identification of characteristic peaks by matching MS and MS/MS mass spectrometry information with metabolite databases. The MS mass error was set to less than 10 ppm, and metabolites were identified based on MS/MS matching scores. The primary databases included mainstream public databases such as http://www.hmdb.ca/ (accessed on 23 September 2024) and https://metlin.scripps.edu/ (accessed on 25 September 2024), as well as self-constructed databases.

### 2.8. Statistical Analysis

Data on pH values, viable bacterial counts, and sensory scores were expressed as “mean ± standard deviation (SD)”. One-way analysis of variance (ANOVA) with a 95% confidence interval was performed, followed by multiple comparisons using Duncan’s method, and these statistical analyses were completed using the SPSS 21.0 statistical software package (IBM; https://www.ibm.com/analytics. accessed on 15 March 2025). Multivariate statistical analyses, including data transformation, Pareto scaling, principal component analysis (PCA), and heatmap visualization, were carried out using the statistical analysis module of MetaboAnalyst 5.0 (https://www.metaboanalyst.ca. accessed on 3 April 2025). Non-volatile metabolic features with variable importance in projection (VIP) values > 1 or <−1 and *p* < 0.05 were identified as differential metabolites. Metabolic pathway analysis was conducted based on online databases: the Kyoto Encyclopedia of Genes and Genomes (KEGG, https://www.genome.jp/kegg. accessed on 23 April 2025) and the Human Metabolome Database (HMDB). Through pathway topology analysis based on relative betweenness centrality, pathways with both low *p*-values and high Impact Values were determined as key pathways.

## 3. Results and Discussion

### 3.1. The Effect of Single and Co-Fermentation of L. sakei and S. carnosus on the pH Value and Viable Cell Count of Fermented Meat Products

**Figure 1:**
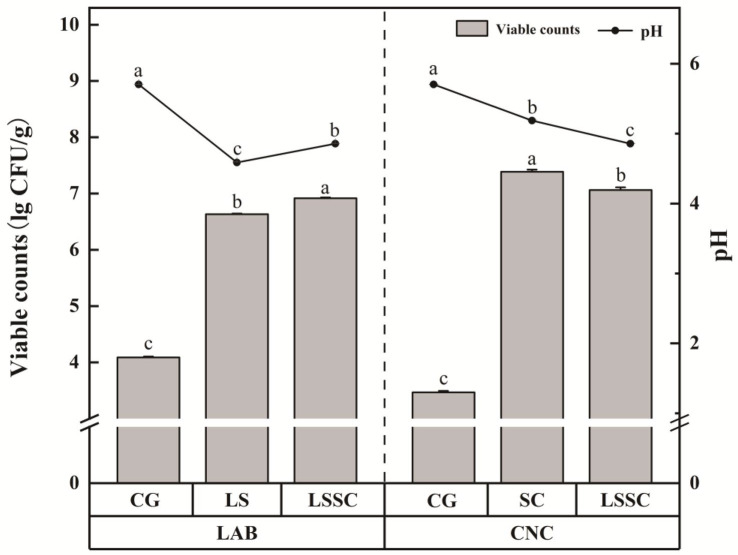
The pH and viable cell count of non-fermented and different strain fermentation groups. LAB and CNC represent the viable cell counts of lactic acid bacteria and staphylococci. The data are expressed as the means ± standard deviations (*n* = 3). Different lowercase letters indicate significant differences (Duncan’s test, *p* < 0.05).

Compared to the CG group, the pH values of *L. sakei* and *S. carnosus* single and co-fermented sausages decreased after 48 h (*p* < 0.05): CG group: 5.70; LS group: 4.59; SC group: 5.19; LSSC group: 4.86. The results indicate that *S. carnosus* has acid-producing abilities during single fermentation (*p* < 0.05), but the effect is not as strong as that of *L. sakei* (Figure 1).

After 48 h of fermentation, both lactic acid bacteria and staphylococci significantly increased (*p* < 0.05). The viable cell counts of *S. carnosus* in single fermentation increased from the initial inoculum (7.2 lg CFU/g) to 7.4 lg CFU/g after 48 h. In contrast, the viable cell counts of *L. sakei* in single fermentation decreased from the initial inoculum (7.1 lg CFU/g) to 6.6 lg CFU/g after 48 h, as LAB rapidly acidify the substrate by synthesizing organic acids, inhibiting their own growth [17]. The results suggest that both *L. sakei* and *S. carnosus* can adapt to the meat environment. An interesting phenomenon was observed during the co-fermentation of *L. sakei* and *S. carnosus*. Due to the excellent acid-producing ability of *L. sakei*, both its own growth and that of *S. carnosus* were inhibited during fermentation. In the LSSC group, the inoculum of *S. carnosus* (7.2 lg CFU/g) was the same as in the SC group, but the inoculum of *L. sakei* (6.6 lg CFU/g) was reduced threefold. After 48 h of fermentation, the viable cell counts of lactic acid bacteria and staphylococci in the LSSC group were 6.9 lg CFU/g and 7.1 lg CFU/g, respectively, with a single fermentation; two bacteria showed a trend of growth in the opposite direction. Besides environmental factors (pH and temperature), these results suggest that there is an interaction between *L. sakei* and *S. carnosus*. During the fermentation process, co-cultivation with *S. carnosus* significantly promoted the growth of *L. sakei*. However, the growth of *S. carnosus* was simultaneously affected by co-cultivation with *L. sakei*. Studies have shown that the microbial community and metabolites in fermented sausages are correlated. The above findings suggest that co-fermentation of *L. sakei* and *S. carnosus* produces certain substances that are conducive to the growth of lactic acid bacteria [18], which requires further validation through metabolomics analysis.

### 3.2. The Effect of Single and Co-Fermentation of L. sakei and S. carnosus on the Metabolomics of Fermented Meat Products

**Figure 2:**
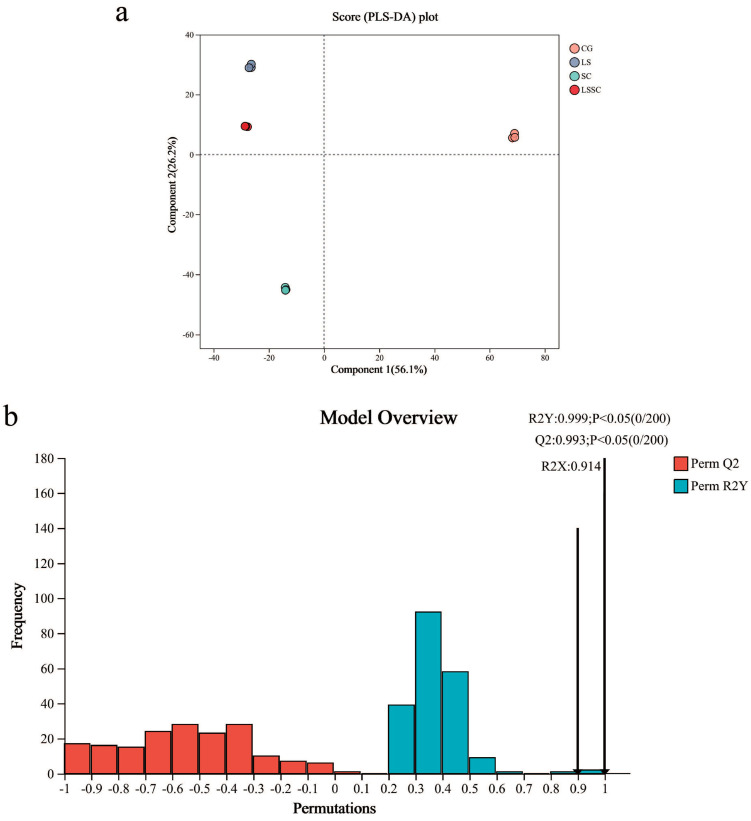
Partial Least Squares Discriminant Analysis (PLS-DA) plot of the non-volatile metabolite profiles of non-fermented and different strain fermentation groups (**a**) and model verification for the OPLS-DA model (**b**).

PLS-DA (Partial Least Squares Discriminant Analysis) analysis was conducted for the non-volatile components of the four groups (CG, LS, SC, and LSSC) (Figure 2a). From the PLS-DA Score plot, the degree of clustering and dispersion of the samples could be perceived. The nearer the sample distribution points were to each other, the more alike the composition and concentration of variables or molecules in those samples were. On the contrary, the farther apart the sample points were, the greater the disparity was. The significant separation of the four groups in the plot indicated a distinct and effective classification. The OPLS-DA (Orthogonal Partial Least Squares Discriminant Analysis) models were backed by high values of R2Y and Q2 (R2Y = 0.999, Q2 = 0.993) (Figure 2b), suggesting that the models had good accuracy and predictability. With the addition of probiotics and fermentation, significant changes in metabolites were observed compared to the CG group. The LS group was closer to the LSSC group than to the SC group, indicating that the molecular composition of the LS and LSSC groups is similar. This suggests that *L. sakei* plays a dominant role during the co-fermentation with *S. carnosus*.

### 3.3. The Changes in Differential Metabolites During Single and Co-Fermentation of L. sakei and S. carnosus

**Figure 3:**
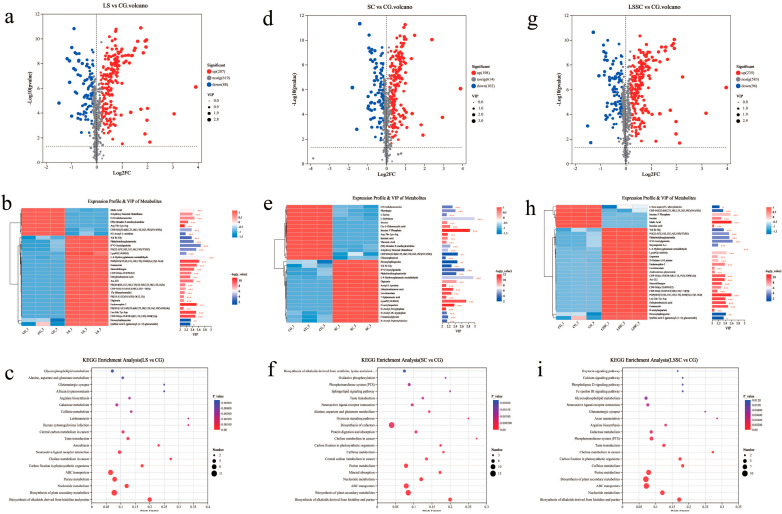
The volcano plots of differential metabolites in pairwise comparisons of LS and CG (**a**), SC and CG (**d**), and LSSC and CG (**g**); the clustering heatmap and VIP bar chart analysis of the top 30 significant differential metabolites of LS and CG (**b**), SC and CG (**e**), and LSSC and CG (**h**); the KEGG pathway differential abundance score plots of the differential metabolites of LS and CG (**c**), SC and CG (**f**), and LSSC and CG (**i**). * indicates *p* < 0.05, ** indicates *p* < 0.01, *** indicates *p* < 0.001.

To comprehensively expose the differential metabolites subsequent to the single and co-fermentation of *L. sakei* and *S. carnosus*, a multi-factor analysis was implemented based on the VIP values obtained from the OPLS-DA model. The differential metabolites in the comparisons between LS and CG, SC and CG, as well as LSSC and CG, were selected by applying VIP > 1, *p* < 0.05, and fold change (>1 or <−1). To enhance the visualization of the variations in differential metabolites, a volcano plot was generated (Figure 3a,d,g). The color of the scatter points signified the ultimate screening outcomes, where red denoted significantly elevated metabolites, blue indicated significantly reduced metabolites, and gray represented metabolites with no significant difference. The three fermented sausage products containing distinct probiotics manifested strikingly dissimilar dispersed distributions and clustering, signifying that the probiotic species exerted a crucial influence on the expression of differential metabolites. We identified 295 differential metabolites between LS and CG, with 207 being upregulated and 88 being downregulated. Between SC and CG, there were 300 differential metabolites, with 198 being upregulated and 102 being downregulated. Between LSSC and CG, there were 331 differential metabolites, with 235 being upregulated and 96 being downregulated. The incorporation of probiotics facilitated the accumulation of metabolites.

Further analysis of the top 30 differential metabolites based on VIP values revealed the following (Figure 3,b,e,h): Between LS and CG, 23 metabolites were upregulated, and 7 metabolites were downregulated. Between SC and CG, 16 metabolites were upregulated, and 14 metabolites were downregulated. Between LSSC and CG, 24 metabolites were upregulated, and 6 metabolites were downregulated. These metabolites primarily involve organic acids and their derivatives, lipids and lipid molecules, as well as nucleosides, nucleotides, and their analogs. By comparing the three groups, we identified several common substances that exhibited consistent trends. Specifically, L-4-Hydroxyglutamate semialdehyde, LysoPC (14:0/0:0), digitonin, and dehydroeburicoic acid showed an upregulated pattern. L-4-Hydroxyglutamate semialdehyde is a derivative of glutamate semialdehyde, with a hydroxyl group introduced at the 4-position of L-glutamic 5-semialdehyde. This compound functions as a metabolite in yeast, mice, and humans, and is functionally associated with L-glutamic 5-semialdehyde, playing a role in arginine and proline metabolism. Digitonin, a glycoside extracted from Digitalis (commonly known as foxglove), increases cell membrane permeability by binding to cholesterol molecules and has been reported to inhibit tumor growth. Dehydroeburicoic acid exerts antitumor, anti-inflammatory, and antidiabetic effects, potentially through the induction of cell apoptosis via the caspase-3 pathway [19]. Research shows that lipid signaling is commonly used as a nutritional substance for microbial growth during fermentation [20]. CDP-DG (22:6 (4Z, 7Z, 10Z, 13Z, 16Z, 19Z)/18:1 (9Z)) shows a downregulation trend and is involved in glycerophospholipid metabolism. Among the differential metabolites in the single fermentation of *L. sakei*, PS (20:4 (8Z, 11Z, 14Z, 17Z)/22:5 (4Z, 7Z, 10Z, 13Z, 16Z)) shows an upregulation trend, also participating in glycerophospholipid metabolism. The downregulation of CDP-DG (22:6 (4Z, 7Z, 10Z, 13Z, 16Z, 19Z)/18:1 (9Z)) can promote the formation of PS (20:4 (8Z, 11Z, 14Z, 17Z)/22:5 (4Z, 7Z, 10Z, 13Z, 16Z)) and LysoPC (14:0/0:0). N2-Acetyl-L-ornithine shows a downregulation trend and is an intermediate in the enzymatic biosynthesis from L-glutamate to L-arginine, involved in arginine biosynthesis. The same substances found in the single fermentation of *L. sakei* and *S. carnosus* include lipids and lipid molecules such as (9E)-Octadec-9-enedioylcarnitine, organic acids and their derivatives like Arg-Thr-Lys-Arg, 4-hydroxy Nonenal Glutathione, and O-ureidohomoserine, all of which show a downregulation trend. The same substances with the same trend are also observed in the single fermentation of *L. sakei* and the co-fermentation with *S. carnosus*. Malic acid is a key compound in the citric acid cycle and contributes a sour taste. Its downregulation can improve flavor. Studies have shown that lactic acid bacteria are capable of conducting malolactic fermentation (MLF). During this process, L-malic acid is enzymatically decarboxylated to produce L-lactic acid and carbon dioxide. This reaction leads to a reduction in total acidity, an increase in pH, improved microbial stability, and enhanced aroma profiles of food products [21]. Furthermore, 11 substances were found to exhibit an increasing trend, including Endomorphin-2 (EM-2), stercobilinogen, emamectin, 3beta-O-(4-amino-4,6-dideoxy-beta-D-galactopyranosyl) digitoxigenin (Asi-222), and CDP-DG(a-25:0/PGE2), among others. Notably, EM-2 is an endogenous opioid peptide that exhibits high affinity and selectivity for the μ-opioid receptor (μ-OR). It demonstrates potent analgesic effects while minimizing adverse reactions such as diminished reward response and respiratory depression following either central or systemic administration. Therefore, EM-2 is considered a promising alternative analgesic with a more favorable safety profile [22,23]. Stercobilinogen is a metabolic product of bilirubin and a precursor in the biosynthesis of stercobilin, which contributes to stool color. Emamectin is a series of macrocyclic lactone derivatives with potent antiparasitic properties. Asi-222 is a glycosylated galactose composed of two galactose units linked by a β-(1->4) bond and is a semi-synthetic cardiac glycoside [24].

In the single fermentation of *S. carnosus*, the differential metabolites are primarily organic acids and their derivatives, along with organic oxygen-containing compounds. Among the organic acids and their derivatives, acetyl-L-tyrosine, N-acetyl-D-tryptophan, and cinnamoylglycine show an upregulation trend, while Cis-3-chloroacrylic acid and L-serine show a downregulation trend; N-acetyl-L-tyrosine acts as a precursor for tyrosine. This compound functions as an inhibitor of EC 2.1.1.4 (acetylserotonin O-methyltransferase) and serves both as a biomarker and a metabolite present in human urine. N-Acetyl-D-tryptophan is an N-acetylated amino acid, specifically the N-acetyl derivative of tryptophan, which participates in various biochemical processes as a metabolite [25]. Cinnamoylglycine belongs to the class of N-acylglycines, with the acyl group characterized as (2E)-3-phenylprop-2-enoyl (cinnamoyl), and primarily functions as a metabolite. L-serine, a recognized non-essential amino acid, serves as a key precursor in the synthesis of intracellular biomolecules, including purines, pyrimidines, and phospholipids. Moreover, it plays a critical role in the development and function of the central nervous system [26,27]. The organic oxygen-containing compounds L-sorbinose and threonic acid are downregulated. L-sorbinose is a naturally occurring, low-molecular-weight monosaccharide that exhibits sweetness comparable to sucrose. It also serves as an intermediate in the biosynthesis of vitamin C. Threonic acid is commonly used as a derivative of vitamin C and functions as an antioxidant. The same differential metabolites produced during the single fermentation of *S. carnosus* and the co-fermentation with *L. sakei* include inosinic acid, inosine 5′-phosphate, and inosine, all of which are involved in purine metabolism and show a downregulation trend. Research has shown that the catabolism of inosine is emphasized in coagulase-negative staphylococci, which suggests that *S. carnosus* retains this characteristic during both single fermentation and co-fermentation with *L. sakei*. The unique differential metabolites produced during the co-fermentation of *L. sakei* and *S. carnosus* include soyasaponin A-c, androsterone glucuronide, L-beta-aspartyl-L-phenylalanine, and D-gulono-1,4-lactone. Androsterone glucuronide is a steroid glucosiduronic acid in which androsterone serves as the steroid component. It functions as a metabolite in both humans and mice [28]. D-Gulono-1,4-lactone acts as a substrate for L-gulono-1,4-lactone oxidoreductase (EC 1.1.3.8), an enzyme that catalyzes the final step of vitamin C biosynthesis in both plants and animals [29]. The top 30 differential metabolites based on VIP values from the three groups show that the differential metabolites in the co-fermentation of *L. sakei* and *S. carnosus* are strongly correlated with those in the single fermentation. At the same time, some characteristics of the single-species fermentation are retained.

In addition, the pathways significantly enriched with metabolites from the metabolic set were identified using the hypergeometric distribution algorithm (Figure 3c,f,i). A pathway was considered significantly enriched if the BH-corrected *p* < 0.05. The horizontal axis represents the Difference Abundance Score (DA Score), while the vertical axis indicates the names of KEGG metabolic pathways. The DA Score reflects the overall alteration of all metabolites within a metabolic pathway. A score of 1 implies that the expression trend of all annotated differential metabolites in the pathway is upregulated, and a score of −1 suggests that the expression trend of all annotated differential metabolites in the pathway is downregulated. The length of the line segment represents the absolute value of the DA Score. The size of the dot represents the quantity of annotated differential metabolites in the pathway, with a larger dot signifying a greater number of differential metabolites. The distribution of dots on the right side of the central axis, accompanied by a longer line segment, suggests that the overall expression trend of the pathway is biased toward upregulation. Conversely, a predominance of dots on the left side of the central axis, along with an extended line segment, indicates a tendency toward downregulation. Amino acid, lipid, carbohydrate, and purine metabolism represent the major metabolic pathways throughout the fermentation process. As seen from the figure, in the single fermentation of *L. sakei*, the amino acid metabolism pathway includes alanine, aspartate, and glutamate metabolism and arginine biosynthesis, with most of the differential metabolites in these pathways showing a downregulation trend. Research suggests that carbohydrate metabolism in LAB during food fermentation is often limited by acidification and low pH. Glutamine, glutamate, and arginine play key roles in maintaining pH homeostasis and survival during the stable phase of LAB [30]. The downregulation of differential metabolites in alanine, aspartate, and glutamate metabolism and arginine biosynthesis would contribute to the acid tolerance of *L. sakei*. In lipid metabolism, the overall expression of glycerophospholipid metabolism tends to be upregulated. Wang et al. [3] found that during the mixed fermentation of lactic acid bacteria (LAB) and coagulase-negative staphylococci (CNS) in tilapia sausage, glycerophospholipid metabolism emerged as a dominant metabolic pathway. Experimental evidence demonstrated that this mixed fermentation induced the production of phospholipase, which subsequently promoted the hydrolysis of glycerophospholipids. In addition, galactose metabolism showed a general trend of upregulation during the fermentation process. Studies have shown that all organisms require purines to function and that certain LAB can absorb purines [31]. In purine metabolism, the overall expression tends to be downregulated. During the single fermentation of *S. carnosus*, alanine, aspartate, and glutamate metabolism show stable expression, while purine metabolism overall tends to be downregulated. In the co-fermentation of *L. sakei* and *S. carnosus*, arginine biosynthesis shows a downregulation trend, glycerophospholipid metabolism and galactose metabolism show upregulation trends, and purine metabolism shows a downregulation trend. We can observe that the co-fermentation of *L. sakei* and *S. carnosus* shows similar pathway enrichment patterns to the single fermentation of *L. sakei*. During the sausage fermentation process, LAB dominates over CNS.

### 3.4. Consistent Differential Metabolites Identified After the Single and Co-Fermentation of L. sakei and S. carnosus

**Figure 4:**
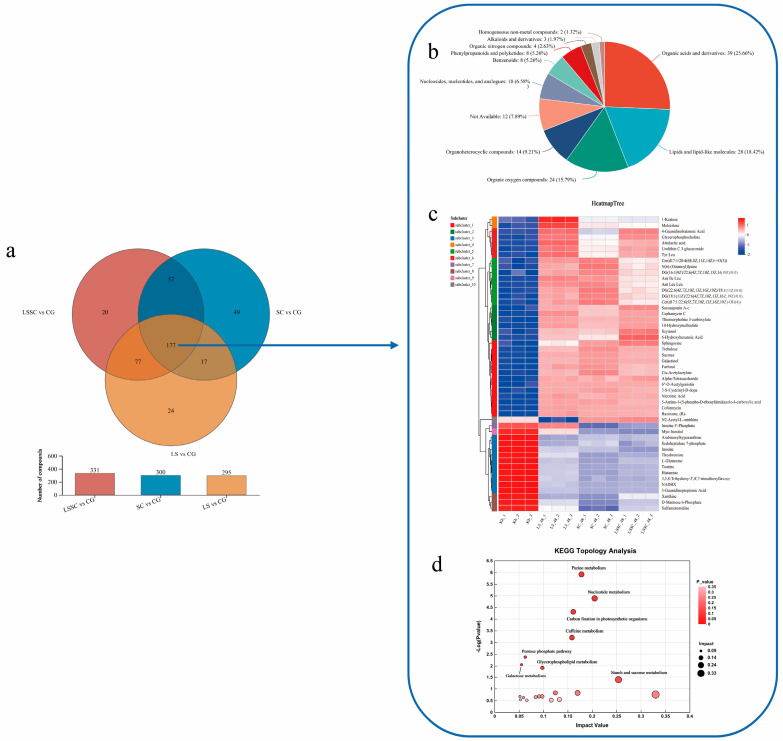
The Venn diagram shows the common or unique differential metabolites between the single and co-fermentation of *L. sakei* and *S. carnosus* (**a**). The HMDB compound classification of the common differential metabolites between the single and co-fermentation of *L. sakei* and *S. carnosus* (**b**). The clustering heatmap analysis of the common differential metabolites (**c**). The KEGG pathway analysis of the common differential metabolites (**d**).

To enhance data visualization, a Venn diagram of the differentially expressed metabolites after screening was created. Compared to the CG group, 295, 300, and 331 differential metabolites were detected in LS, SC, and LSSC, respectively. A total of 177 common metabolites were identified across the three groups (Figure 4a). We further analyzed these 177 common differential metabolites and matched them with the Human Metabolome Database (HMDB), classifying them into 11 different categories. The main categories included organic acids and derivatives (25.66%), lipids and lipid-like molecules (18.42%), organic oxygen-containing compounds (15.79%), and organic heterocyclic compounds (9.21%) (Figure 4b).

Additionally, hierarchical clustering analysis was performed on the 177 differentially expressed metabolites, with the top 50 metabolites shown in Figure 4c. From the figure, a clear trend of significant upregulation and downregulation of differential metabolites can be observed. The expression levels of metabolites were more upregulated in the single fermentation of *L. sakei* compared to *S. carnosus* single fermentation and the co-fermentation of both bacteria. Among these, 1-kestose and melezitose exhibited the most significant changes. 1-kestose is a non-reducing trisaccharide formed by the condensation of sucrose and fructose via (2,6)- and (2,1)-linkages. Melezitose is a non-reducing trisaccharide that can be partially hydrolyzed into glucose and laminaribiose. Furthermore, concerning N2-acetyl-L-ornithine, its concentration is elevated in the LSSC and SC groups compared to the CG group, while it is significantly reduced in the LS group relative to the CG group. N2-acetyl-L-ornithine serves as an intermediate in the enzyme-catalyzed biosynthesis pathway from L-glutamate to L-arginine. These compounds may act as biomarkers for the single fermentation process of *L. sakei*. In sub-cluster 6, 4-guanidinobutanoic acid, glycerophosphocholine, atrolactic acid, urolithin C 3-glucuronide, and Tyr Leu are expressed at higher levels in both the LS and LSSC groups when compared to the SC group. Specifically, 4-guanidinobutanoic acid plays a role in arginine and proline metabolism, glycerophosphocholine is involved in glycerophospholipid metabolism, and atrolactic acid participates in tyrosine metabolism. Studies suggest that LAB-mediated arginine conversion in food can lead to spoilage or enhance flavor, and it also affects glycerophospholipid and tyrosine metabolism [32,33]. Urolithin C 3-glucuronide is a metabolite of a class of compounds known as polyphenols. It is a bioactive compound with anti-inflammatory, antioxidant, and anticancer properties. The specific expression of these compounds in the LS and LSSC groups may be triggered by the addition of *L. sakei*. We also noticed that inosine 5′-phosphate (IMP) and myo-inositol were expressed at lower levels in the SC and LSSC groups compared to the LS group. IMP plays a central role in purine metabolism, as de novo purine biosynthesis initiates with the synthesis of IMP. This compound serves as a key precursor that can be converted into all other purine nucleotides. Myo-inositol is involved in galactose metabolism; it is a water-soluble, six-carbon cyclic compound that is essential for the growth of various bacterial, fungal, and yeast species [34]. This trend suggests that *S. carnosus* utilizes these substances more than *L. sakei*, and this characteristic is retained during the co-fermentation of both bacteria. In conclusion, the specific expression trends of N2-acetyl-L-ornithine, IMP, and myo-inositol can serve as a basis for further research into the interactions between *L. sakei* and *S. carnosus*.

KEGG metabolic pathway analysis proves highly beneficial for comprehending potential metabolic pathways (Figure 4d). This analysis can distinguish the effects of various microorganisms on the sausage metabolic pathways. To further clarify the relevant metabolic pathways, KEGG topological analysis was performed on 177 differential metabolites. Each bubble in the graph represents a KEGG Pathway. The horizontal axis indicates the relative importance of metabolites in the pathway, namely the Impact Value. The vertical axis represents the enrichment significance of metabolites in the pathway, namely −log10 (*p* value). The size of the bubble indicates the Impact Value; the larger the bubble, the greater the significance of the pathway. Based on statistical significance (*p* < 0.05) and the impact factor threshold (impact > 0.1), a total of 8 major pathways were selected, including starch and sucrose metabolism, galactose metabolism, pentose phosphate pathway, glycerophospholipid metabolism, caffeine metabolism, purine metabolism, nucleotide metabolism, and carbon fixation in photosynthetic organisms.

### 3.5. Metabolic Pathway Analysis of the Single and Co-Fermentation of L. sakei and S. carnosus

**Figure 5:**
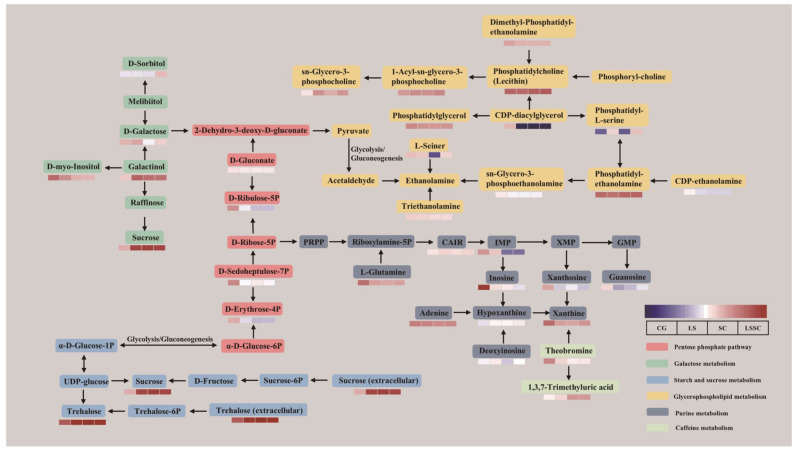
Overview of the metabolic pathways of metabolites during the single and co-fermentation of *L. sakei* and *S. carnosus*.

Based on the KEGG pathway analysis, eight major metabolic pathways were identified. The six pathways that have the most significant impact on beef sausage metabolites during the single and co-fermentation of *L. sakei* and *S. carnosus* are summarized and depicted in Figure 5. The changes in metabolite content during the single and co-fermentation of *L. sakei* and *S. carnosus* (Table S1) and chromatograms of individual microorganisms and co-fermentation finished beef sausages (Figure S1) are in the Supplementary Materials. In the pentose phosphate pathway, the levels of D-ribulose-5P, D-sedoheptulose-7P, and D-erythrose-4P in the LS, SC, and LSSC groups were all lower compared to the CG group, indicating that these substances were utilized during the fermentation process. The pentose phosphate pathway is one of the methods of glucose oxidation and breakdown, where glucose is directly oxidized through dehydrogenation and decarboxylation, bypassing glycolysis and the tricarboxylic acid cycle. Studies have shown that the primary pathway for glucose oxidation and breakdown during the single and co-fermentation of *L. sakei* and *S. carnosus* is the pentose phosphate pathway. Another noteworthy observation is that galactose metabolism and starch and sucrose metabolism can synthesize sucrose through the pentose phosphate pathway, and the sucrose content in the LS, SC, and LSSC groups was significantly higher compared to the CG group. Sucrose can add sweetness to fermented sausages, contributing to the formation of their unique flavor. In the starch and sucrose metabolism pathway, the content of trehalose also increased after fermentation by the strains. Trehalose, a natural sweetener, plays a role in preventing food spoilage, maintaining freshness, and enhancing food quality, which is beneficial for the flavor formation of fermented sausages. Regarding D-galactose, the content in the LS group increased by 0.02-fold compared to the CG group, while in the SC group and LSSC group, the content decreased by 0.16-fold and 0.06-fold, respectively. This indicates that D-galactose produced during the fermentation of *L. sakei* can be utilized by *S. carnosus*. D-Galactose is commonly used as a sugar substitute [35]. Regarding D-sorbitol, its content decreased in the LS and SC groups compared to the CG group, while in the LSSC group, it increased by 0.2-fold compared to the CG group, indicating that the co-fermentation of *L. sakei* and *S. carnosus* promotes the production of D-sorbitol. Purine metabolism is fundamental to nucleic acid synthesis and plant metabolic pathways [36]. Several key purine metabolites, including IMP, inosine, xanthine, guanosine, and xanthosine, showed reduced abundance during the fermentation process. These metabolites are critical for microbial growth and reproduction, and similarities between LAB and CNS have been reported. Previous studies have shown that changes in different metabolic pathways and metabolite levels can influence bacterial proliferation during fermentation. Bacterial proliferation depends on metabolite changes, and this interaction leads to increases or decreases in metabolite levels during fermentation [37]. In glycerophospholipid metabolism, CDP-diacylglycerol (CDP-DAG) is utilized during both the single and co-fermentation of *L. sakei* and *S. carnosus*. CDP-DAG acts as an intermediate at a key branching point in lipid metabolism. It participates in multiple biosynthetic pathways, leading to the formation of various phospholipid end products. In contrast, L-serine is a significant intermediate metabolite in biological systems and serves as a precursor for the synthesis of several compounds, including glycine, nucleotides, choline, and phospholipids. The content of L-serine in the SC group decreased by 0.49-fold compared to the CG group. Phosphatidyl-L-serine is an important membrane phospholipid found in bacteria, yeast, plants, and mammalian cells. The content of phosphatidyl-L-serine in the LS and LSSC groups increased by 0.7 and 0.76-fold, respectively, compared to the CG group. It can be observed that the trend in metabolite expression in glycerophospholipid metabolism is consistent in both the LS and LSSC groups. In caffeine metabolism, theobromine serves as the primary bitter component in chocolate. When compared to the CG group, the content of theobromine in the groups supplemented with strains exhibited varying degrees of decline. This downward tendency has the potential to mitigate bitterness and enhance the flavor of fermented sausages.

## 4. Conclusions

The study shows that after the single and co-fermentation of *L. sakei* and *S. carnosus*, the upregulated metabolites significantly increased compared to pre-fermentation, indicating that the fermentation process promoted the generation of certain substances. These substances primarily involve organic acids and their derivatives, lipids and lipid molecules, as well as nucleosides, nucleotides, and their analogs. We found that the pathway enrichment during the co-fermentation of *L. sakei* and *S. carnosus* was similar to that of *L. sakei* single fermentation, suggesting that LAB dominate over CNS in the sausage fermentation process. By comparing the shared differential metabolites across the three groups and pre-fermentation, the unique expression trends of N2-acetyl-L-ornithine, IMP, and myo-Inositol could serve as a basis for further research on the interactions between *L. sakei* and *S. carnosus*. Based on KEGG topological analysis, eight key metabolic pathways were selected, including purine metabolism, starch and sucrose metabolism, glycerophospholipid metabolism, and caffeine metabolism. Additionally, the metabolic pathways revealed that D-galactose is produced during *L. sakei* fermentation can be utilized by *S. carnosus*, and co-fermentation of *L. sakei* and *S. carnosus* promotes the production of D-sorbitol. These findings demonstrate the interaction between *L. sakei* and *S. carnosus*, and further targeted metabolic verification of individual substances can be conducted in future studies to explore their interactions. Our data support the idea that probiotics can undergo metabolic interactions during sausage fermentation, thereby enhancing the production of bioactive metabolites. Our study offers intriguing perspectives on microbial interactions within industrial fermentation ecosystems.

## Data Availability

Upon reasonable request, and subject to review, the authors will provide the data that support the findings of this study.

## Supplementary Materials

The following supporting information can be downloaded at: https://www.mdpi.com/article/10.3390/microorganisms13071523/s1, Table S1: Changes of metabolite content during the single and co-fermentation of *L. sakei* and *S. carnosus*; Figure S1: Chromatograms of individual micro-organisms and co-fermentation.
