# Supplementary material for: Comparative Effects of the Single and Binary Fermentations of Latilactobacillus sakei and Staphylococcus carnosus on the Growth and Metabolomic Profiles of Fermented Beef Sausages
Source: Microorganisms. 2025 Jun 29;13(7):1523. doi: 10.3390/microorganisms13071523 (PMC12299760; doi:10.3390/microorganisms13071523)
Supplement: Supplementary file 1 [file microorganisms-13-01523-s001.zip › microorganisms-3673377-supplementary.pdf]

Supplementary material

Supplementary table Changes of metabolite content during the single and co-fermentation of *L. sakei and S. carnosus*

Metabolite	Formula	Retention time min	RSD	CG	LS	SC	LSSC
D-Gluconate	$C_6H_{11}O_7$	1.9593	0.0563	5.3770	5.2977	5.2913	5.3180
D-Ribulose-5P	$C_5H_{11}O_8P$	1.1121	0.0303	6.4747	4.9973	4.4867	4.4883
D-Sedoheptulose-7P	$C_7H_{15}O_{10}P$	0.9171	0.0341	6.6460	5.1000	5.3017	5.0720
D-Erythrose-4P	$C_4H_9O_7P$	0.8858	0.0471	6.1033	4.8083	4.4297	4.4790
Sucrose	$C_{12}H_{22}O_{11}$	1.2667	0.0064	6.0480	7.8360	7.9503	7.8140
Trehalose	$C_{12}H_{22}O_{11}$	1.0263	0.0042	7.4483	8.1677	8.2713	8.1980
Galactinol	$C_{12}H_{22}O_{11}$	0.9950	0.0015	3.8703	5.5737	4.9707	4.9860
D-Myo-Inositol	$C_6H_{12}O_6$	0.9171	0.0084	7.1447	6.5097	5.9990	5.9350
D-Sorbitol	$C_6H_{14}O_6$	0.9171	0.0034	4.9227	4.8910	4.8473	5.8840
D-Galactose	$C_6H_{12}O_6$	6.2310	0.0553	5.9857	6.1507	5.0410	5.6130
L-Serine	$C_3H_7NO_3$	0.9501	0.0143	5.8340	5.7363	3.0083	5.5617
Phosphatidylcholine (Lecithin)	$C_{42}H_{78}NO_8P$	6.6541	0.0453	7.2453	7.1743	7.3680	7.4410
CDP-diacylglycerol	$C_{52}H_{83}N_3O_{15}P_2$	3.0207	0.1172	5.9073	2.1013	1.9933	2.0777
Phosphatidylglycerol	$C_{35}H_{69}O_{10}P$	7.2043	0.0488	6.5507	6.4433	6.3880	6.4223
Phosphatidyl-ethanolamine	$C_{43}H_{78}NO_7P$	7.7289	0.0213	7.1563	7.0203	7.2550	7.2857
CDP-ethanolamine	$C_{11}H_{20}N_4O_{11}P_2$	3.2447	0.0339	5.2440	4.8030	4.7247	4.7187
sn-Glycero-3-phosphocholine	$C_8H_{20}NO_6P$	0.8946	0.0132	5.3990	6.4580	6.0573	6.4023
Sn-glycero-3-Phosphoethanolamine	$C_5H_{14}NO_6P$	3.2447	0.0266	5.3407	5.0287	5.0183	5.0053
Phosphatidyl-L-serine	$C_{41}H_{76}NO_{10}P$	2.8458	0.1339	3.2830	5.5617	3.1607	5.7697
1-Acyl-sn-glycero-3-phosphocholine	$C_{28}H_{48}NO_7P$	6.9017	0.0265	6.3377	6.5233	6.4713	6.6010

Dimethyl-Phosphatidyl-ethanolamine	$C_4H_{11}NO$	7.7368	0.0146	6.1770	6.0157	5.9787	5.9860
Triethanolamine	$C_6H_{15}NO_3$	7.6251	0.0232	5.6243	5.5870	5.4973	5.5023
L-Glutamine	$C_5H_{10}N_2O_3$	0.9741	0.0071	7.0587	6.2453	6.1277	6.2307
IMP	$C_{10}H_{13}N_4O_8P$	1.3305	0.0020	6.4050	5.7497	3.5097	3.5080
Adenine	$C_5H_5N_5$	7.0930	0.0131	6.7747	6.6743	6.5793	6.5913
Hypoxanthine	$C_5H_4N_4O$	1.5327	0.0512	4.8567	5.2063	5.2093	5.3030
Inosine	$C_{10}H_{12}N_4O_5$	3.0361	0.0018	8.1010	5.4570	5.2997	4.8627
Xanthine	$C_5H_4N_4O_2$	1.7270	0.0056	7.1140	6.2550	6.1700	6.4453
Guanosine	$C_{10}H_{13}N_5O_5$	3.0361	0.0359	5.5483	4.1513	4.4737	4.9030
Xanthosine	$C_{10}H_{12}N_4O_6$	3.1609	0.0277	6.1523	4.6433	4.9863	4.5457
CAIR	$C_9H_{14}N_3O_9P$	0.9820	0.0106	5.3887	5.5597	5.4647	5.5590
Deoxyinosine	$C_{10}H_{12}N_4O_4$	3.2233	0.0150	5.0337	5.3007	4.6050	5.1450
Theobromine	$C_7H_8N_4O_2$	0.8858	0.0082	6.6500	5.5787	5.2847	5.3557
1,3,7-Trimethyluric Acid	$C_8H_{10}N_4O_3$	1.0886	0.0128	5.2560	5.5280	6.4087	6.3680

RSD: relative standard deviation of QC sample

Supplementary figure

a

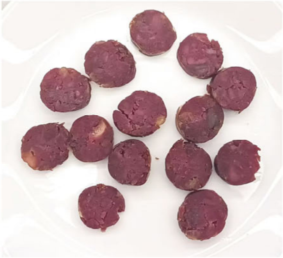

b

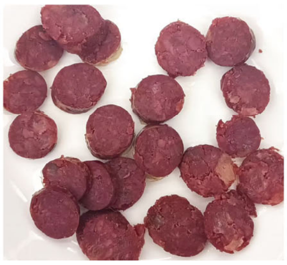

c

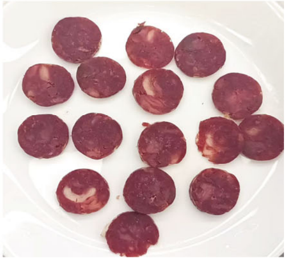

Chromatograms of individual microorganisms and co-fermentation. Finished beef sausages fermented solely by *L. sakei* (a). Finished beef sausages fermented solely by *S. carnosus* (b). Finished product of beef sausage fermented by a combination of *L. sakei* and *S. carnosus* (c)
